# How do undergraduate STEM mentors reflect upon their mentoring experiences in an outreach program engaging K-8 youth?

**DOI:** 10.1186/s40594-017-0057-4

**Published:** 2017-02-10

**Authors:** Kari Nelson, Jaime Sabel, Cory Forbes, Neal Grandgenett, William Tapprich, Christine Cutucache

**Affiliations:** 1 Department of Biology, University of Nebraska at Omaha, Omaha, NE USA; 2 Department of Biological Sciences, The University of Memphis, Memphis, TN USA; 3 School of Natural Resources, University of Nebraska, Lincoln, NE USA; 4 Department of Teacher Education, University of Nebraska at Omaha, Omaha, NE USA

**Keywords:** Mentor, STEM, Undergraduate, Career, Content knowledge, Outreach

## Abstract

**Background:**

Many university students are becoming involved in mentoring programs, yet few studies describe the impact of mentoring on the mentor. Additionally, many studies report that students graduating from college are not prepared to enter the workforce in terms of key career skills and/or content knowledge. Herein, we examine the impact of our program, NE STEM 4U (Nebraska Science, Technology, Engineering and Math for You), in which undergraduate (UG) mentors engage K-8 youth in after-school STEM experiments. The UGs reflected upon their experiences using post-mentoring evaluations, 12- and 24-week interviews, and exit surveys. Many of the questions asked of the mentors related directly to their own professional development, such as self-evaluation of communication, organization, and problem-solving skills, while other questions related to content knowledge and reflection.

**Results:**

Post-mentoring, UGs reflected on the delivery/teaching significantly more (*p* ≤ 0.001 for each) than other variables (i.e., their own content knowledge gains, the students’ content knowledge gains, scaffolding the lessons, or overall professional growth). By analyzing the evaluations and interviews together, some significant, self-reported gains emerged. For example, 94.15% of the UG reported that the experience was beneficial to their education. Additionally, UG mentors self-reported significant gains (*p* ≤ 0.01 for each) moving from 12- to 24-weeks in the program in the categories of organization, STEM content knowledge, preparedness to teach, and engagement in the program. However, UG did not report significant gains in dependability. Importantly, when mentors ranked themselves at 24-weeks, they were blinded to (unaware of) the ranking they gave themselves at 12-weeks.

**Conclusions:**

This study helps to fill a gap in the literature by providing insight into the gains UG mentors report attaining after mentoring to K-8 students. These data suggest that participation by UGs in this program promoted self-reflection as well as self-reported gains related to career preparedness and STEM content knowledge.

**Electronic supplementary material:**

The online version of this article (doi:10.1186/s40594-017-0057-4) contains supplementary material, which is available to authorized users.

## Background

Volunteer tutoring or mentoring programs that pair undergraduate (UG) students with K-8 students have been shown to improve academic skills for tutored students (e.g., Ritter et al. 2009), but few studies have examined the effects on the UG tutors themselves (Carpenter, 2015). Moreover, many of the past studies have focused on mentoring programs that emphasize math or reading, rather than science. Studies that examine how UG mentors think about and teach life science concepts to younger students could help to create a better understanding of the ideas that UGs have about life science concepts, how they integrate new knowledge they are learning from college coursework into the more elementary concepts they are teaching, and how engaging in these ideas helps them to develop as disciplinary thinkers.

By serving as tutors to younger students, the UG mentors act as “the more knowledgeable other” that is required for the zone of proximal development (Vygotsky 1934; Vygotsky 1986). They must decide on the scaffolds they need to use to help the younger students understand the material (Hmelo-Silver et al. 2007). In addition, by engaging with younger students in this way, UG mentors participate in a co-constructed zone of proximal development, in which the mentors learn from the students’ ideas as they help advance students’ understanding (Ash & Levitt 2003). Further, how UGs reflect on their mentoring experiences, and the content they taught, can inform the design of mentoring programs, particularly the reflective components of those programs, in order to ensure academic benefit for the UGs as well as the students they are mentoring. In this way, prompts for reflection after teaching according to Lin et al. (1999) will promote “active monitoring, evaluating and modifying (of) one’s thinking” (p. 43) to help UG mentors make sense of the experience, problem solve, and adapt to different teaching (and learning) environments (Bruer 1993). Additionally, promoting self-evaluation after mentoring can encourage the UGs to consider both their own content knowledge and how to best support younger students in life science lessons (Phillips & Bond 2004).

### Research questions

In order to fill this gap in the literature, the current study was designed to examine UG mentors’ experiences as they engaged with mentoring life science lessons in an outreach program, utilizing reflection prompts to encourage UG mentors to evaluate their mentoring experiences. Specifically, this study is informed by the following research questions:

1. In what ways does an after-school outreach mentoring program for K-8 students affect UG mentors in terms of personal development, as evidenced by professional preparation and academic/content gains?
2. What factors do UG mentors consider when they evaluate their experiences in an after-school outreach mentoring program for K-8 students?

### Literature review

There is a growing concern that the number of well-educated professionals in science, technology, engineering, and mathematics (the “STEM” fields) is far fewer than needed, establishing a kind of “global race” for building the STEM pipeline (The Observatory 2013). While this trend is evident in many countries, this literature review is primarily from the perspective of the USA. Reports, such as 2007 *Rising Above the Gathering Storm* and 2010 *Rising Above the Gathering Storm*, *Revisited* from the USA, indicate a critical need to meet and enhance STEM standards (Augustine et al. 2010). These publications highlight a growing competitiveness among countries. At the same time, occupational projections, in the USA alone, predict a need for several million new college graduates with STEM degrees by 2018 (Carneval et al. 2010; Chen & Soldner 2013; STEM Connector Report 2014). Furthermore, publications such as *Vision and Change in UG Biology Education* (AAAS 2011; Brewer & Smith 2011), the Discipline-Based Educational Research (DBER) Report (Singer et al. 2012), and the 2015 Employer Survey from the National Association of Colleges and Employers (NACE) *Job Outlook* publication (2014) all suggest a need to improve pre-professional training for STEM UGs if they are to be competitive job applicants that progressively contribute to the economy (Langdon et al. 2011). For example, the US Department of Commerce concluded that future earnings of individuals in STEM fields are, on average, 26% higher than salaries of their peers in non-STEM fields (U.S. Department of Commerce, 2011). By all accounts, the economic and societal benefits of meeting the STEM challenge are substantial and may well be a major economic driver that makes a better life for populations worldwide (New York Academy of Sciences 2014).

To meet these challenges, the retention of existing STEM UGs within college programs is particularly important (NSB 2010). Previously released STEM Attrition Report (ED/IES, 2013), which examines the attrition of college students from STEM fields over 6 years, indicates that 48% of those pursuing a bachelor’s degree and 69% of those pursuing an associate’s degree in STEM majors left these fields of study. Furthermore, approximately one half of the students that left STEM majors switched to non-STEM fields, and the remainder typically exited college prior to earning a degree or certificate (ED/IES 2013).

Beyond the need to retain STEM majors, there exists a growing need for STEM professionals that can productively interface with recent advancements that cross both science and technology. These advancements have radically changed not only the application of science but also STEM learning and the professional fields associated with that learning. As outlined in the Vision and Change report (AAAS 2011), the dynamic and interdisciplinary STEM environment of the twenty-first century requires that scientists not only understand core disciplinary concepts but also use critical thinking, communication, reflection, and reasoning skills to translate those concepts to real-life solutions. In turn, UG education must change to ensure that students understand the core concepts and also develop the core competencies necessary to succeed in today’s STEM professions.

Employers from various professions, including STEM and non-STEM areas, recognize the importance of developing core professional skills (i.e., communication, problem-solving, critical thinking, and teamwork). Recently, a survey of employers reported that many college graduates lack the leadership and organizational skills they need to succeed in the workplace (Dostis 2013). Additionally, in the current NACE *Job Outlook* publication (2014), over 70% of the employers participating in the survey seek attributes of leadership, teamwork, a strong work ethic/dependability, and communication skills (written and verbal) in their future employees. In light of these recent reports, it is imperative to capitalize on practices and methods that successfully develop a well-trained and prepared STEM workforce.

While innovative and engaging STEM education has the potential to prepare students to be successful contributors in the workplace, too often, STEM classrooms are dominated by traditional, transmittal lecture formats. This teaching style is often viewed as necessary for delivery of heavy content loads in STEM courses. Many faculty feel that they must “cover all of the material.” It has been well documented that this type of traditional lecture does not increase critical thinking or problem-solving skills (Aguirre et al. 2013; Rabe-Hemp et al. 2009; Tiwari et al. 2006). While hands-on laboratories and their instruction can support content knowledge and expand problem-solving skills, labs are often prescribed in nature, thereby falling short of fostering critical thinking (Cooper et al. 2012; Dolan 2012; Hmelo-Silver 2004). In contrast, active-learning strategies where UGs are involved in research, teaching, and mentoring enhance the UG experience and build a community prepared for graduate schools, professional schools, or the workforce (Karukstis & Hensel, 2010). These instructional approaches also help students to learn and retain complex concepts (Avanzato 2000).

While these techniques are said to improve undergraduate education, little research has been done to understand the value of mentoring for the mentor (Carpenter 2015). Malone et al. (2002) examined the effects on UGs tutoring elementary students and found changes in UGs’ perspectives, including their identity and personal development, as well as on teaching and learning. Many of the UGs reported that the tutoring experience helped to reinforce academic content learned previously. Similarly, they learned from their tutees as they helped those students to learn (Malone et al., 2002). However, Malone et al. (2002) focused on UGs who were considering a career in teaching and the tutoring was part of a service learning component of an education course; therefore, the academic content focus was regarding teaching methods, scaffolding lessons, and concepts. Other programs have examined how UG mentors impacted high school students in their pursuit of STEM careers (e.g., Marable 1999); however, the effect the experience had on the UG mentors was not examined.

Peer tutoring has also been a focus of past research and has been shown to help support tutors’ own academic learning (Roscoe & Chi 2007; Roscoe & Chi 2008). This academic learning typically occurred through self-monitoring of comprehension, integrating new knowledge with prior knowledge, and in constructing and elaborating knowledge (Roscoe & Chi 2007). However, peer tutors usually focused on delivering knowledge to their tutee rather than on developing their own knowledge (Roscoe & Chi 2007). Tutors were more likely to build knowledge and engage in metacognition of their own ideas when tutees asked them questions that required an inferential answer (Roscoe & Chi 2008).

While some evidence suggests that tutoring or mentoring other students can help the academic learning and confidence of UG mentors (Rao et al. 2007), as well as professional skill development, such as communication, organization, and teamwork (Grant et al. 2015), more work is needed to determine the effects mentoring has on the UG mentors (Carpenter 2015). This is becoming increasingly important as more STEM-related departments are increasingly developing outreach programs to primary and secondary schools (James et al. 2006; Tanner et al. 2003; Williams 2002). It will be important to investigate the impacts on the UGs mentoring younger STEM audiences as such programs become more prevalent.

## Methods

### Intervention: pre-professional training under an outreach program platform

The model we created to address these growing STEM challenges and calls for action in the improvement of STEM education is called NE STEM 4U (Cutucache et al. 2016). This program is a student-run, faculty-supervised program that provides inquiry-based after-school STEM activities for socioeconomically disadvantaged youth in grade K-8 in the Omaha (NE) Public Schools (OPS). Most UG students in the NE STEM 4U program are volunteers (herein referred to as mentors) from disciplinary STEM or professional education departments at the University of Nebraska at Omaha (UNO). Some mentors in leadership positions, such as student officers, are supported by modest stipends. The program incorporates several key practices and methods that contribute to retention of UG students and preparation of a well-trained STEM workforce mentioned above. For example, we use problem-based learning (PBL) as our model of instruction. For the students who are instructed using PBL, it has been shown to improve critical thinking and social skills, increases aptitude, enhances mastery of subject matter, and improves retention of information (Chng et al. 2011; Nicholl & Lou 2012; Salinitri et al. 2012; Wiznia et al. 2012).

**Fig. 1:**
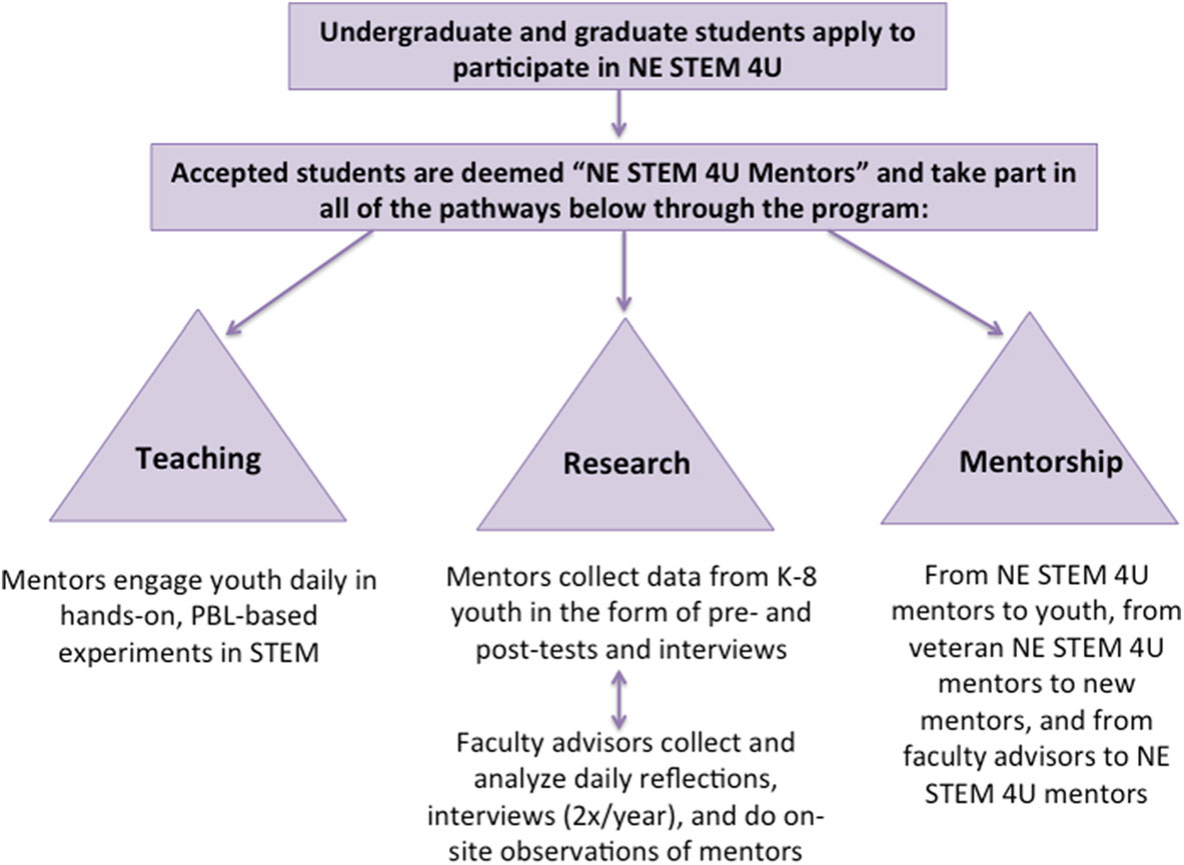
Flow chart of the structure of the NE STEM 4U program

Our model for pre-professional training includes a threefold approach involving research, teaching, and mentoring (Fig. 1). Here, we assess the impact of the NE STEM 4U program on the UG mentor participants using several sources of self-reported data. The self-reported data include post-mentoring surveys, interviews, and end of program surveys.

### Research approach and context

The outreach program, NE STEM 4U, pairs UG and graduate students as STEM mentors with elementary and middle school students. The student mentors provide after-school STEM activities by leading lessons using hands-on activities to middle school students with the aim of providing opportunities for the students to experience disciplinary topics and potentially pursue studies and careers in a STEM area. The program initially began in the spring of 2013 and continues to serve 7–10 Omaha Public Schools per year. It is set up as a pre-professional training program for UG students in that mentors learn how to teach effectively, communicate, conduct research, and provide outreach to area students.

UG mentors teach lessons in the after-school program with themes, such as Forensics or Medicine, that each cover 6-week periods. Each mentor commits at least 4 h per week to prepare topics, design experiments, and teach the lessons to students. Mentors volunteer at the after-school program once per week and typically commit at least 1 year to the program. In order to be accepted as a member of NE STEM 4U, students must submit an application as well as a curriculum vitae or resume, their GPA, and a cover letter that describes their motivation for membership in the organization. To date, the program has had 109 UG mentors. About 40% of the students have been in the program since it started in March of 2013, while the remainder began in August of 2014. The mentors are from a variety of backgrounds and all have an interest in STEM but are not necessarily in a STEM major.

### Participants

From the pool of mentors, selection for inclusion in this specific study was limited to UGs who mentored life science lessons during the fall semester 2013, spring semester 2014, and/or the fall semester 2014. This brought the total number of mentors included in the current study to 18. Demographic information about our mentors related to year in school, major, ethnicity, and gender is presented in Tables 1 and 2. Because only three graduate students participated in this study, their information was pooled with the UGs to protect the identities of participants. All protocols described herein were reviewed and approved through the University of Nebraska Medical Center and the University of Nebraska at Omaha’s Institutional Review Board (IRB#548-12-EX). The consent of the participants was obtained at the beginning of the study; moreover, they were reminded every 12 weeks that their participation in data collection was voluntary and that they could withdraw at any time.

**Table 1:** General demographics for UG student mentors included in this study

	Level		Ethnicity		Gender	
	Undergraduate	Graduate	White	Asian	Male	Female
Number of students	15	3	16	2	9	9
Percent of total	83.3	16.7	88.9	11.1	50	50

**Table 2:** Student characteristics of undergraduate student mentors related to major and college preparation

	Major		1st generation student		Transfer		If transfer, from where	
	Biology	Biotechnology	Y	N	Y	N	CC	4 years
Number of students	10	8	7	11	14	4	10	4
Percent of total	55.6	44.4	38.9	61.1	77.8	22.2	71.4	28.6

### Data collection and analyses

**Fig. 2:**
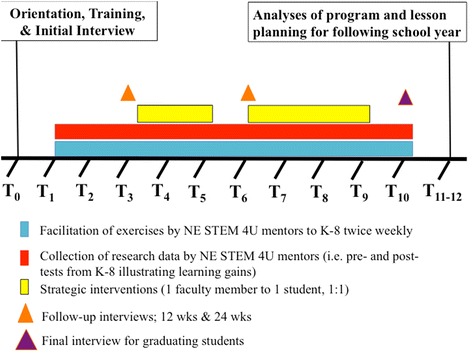
Timeline of data collection during program implementation and assessment representative of an academic year. *T* time as measured in months

For the purpose of this study, mentors were asked about their experiences in three ways: post-mentoring surveys, 12- and 24-week interviews (or first and then second semesters), and a post-program interview. Table 3 includes a summary of each of these instruments. Figure 2 illustrates a timeline of data collection. Each mentor was asked, but not required, to complete a survey after each lesson they taught. Surveys were submitted online through the University of Nebraska at Omaha OrgSync Website (www.orgsync.com) from the fall semester 2013, spring semester 2014 and fall semester 2014. These survey responses were used to examine how the mentors reflected upon their experience in teaching STEM lessons (*n* = 64 total) (Table 3a).

**Table 3:** Questions administered to NE STEM 4U UG and graduate participants regarding their experience in the program

A. Questions from post-mentoring survey	
1. What activity did you participate in and on which date?	
2. Did you find this experience to be beneficial to your education?	
3. Did you feel a sense of accomplishment with helping community members?	
4. What would you do differently next time?	
5. What did you like most about the experience?	
6. What did you like least about the experience?	
7. How do you think this experience most helped the community?	
8. Please provide feedback on your K8 students during this lesson in regards to engagement, comprehension, and other observations.	
9. Other comments:	
B. Questions from 12- and 24-week Time point Interview	
1. Rate your organizational skills	
2. Rate your preparedness skills	
3. Rate your engagement skills (i.e. ability to grab attention through meaningful discussion)	
4. Rate your dependability skills	
5. Rate your communication skills	
6. Can you think of a time recently where you have had to problem solve or think critically in NE STEM? If yes, please describe.	
7. What kind of career do you expect to enter?	
8. Do you plan to include teaching and/or mentoring in your career?	
9. What is one thing that you have liked about the NE STEM 4U program?	
10. What is one thing that you have disliked about the NE STEM 4U program?	
C. Questions from End of Program Survey	
1. Provide your college major(s)	
2. Provide your GPA	
3. What is your career plan?	
4. Do you have an employer or a form of employment already identified?	
5. Have you had any job opportunities as a result of the NE STEM 4U program?	
6. What was (were) the best experience(s) for you in NE STEM 4U and why?	
7. What recommendations do you have to improve NE STEM 4U?	
8. Did you feel as though you were adequately prepared to begin a career after completing your UG major at UNO? What, if any, role did NE STEM 4U play in that level of preparedness?	
9. What did you feel as though you were missing in your UG career at UNO for career and/or preparation for professional school?	
10. Would you be willing to provide feedback about how NE STEM 4U might have helped your career in the next year and in 5 years? If so, please provide the best ongoing contact information for you.	

A. Prompts from post-mentoring survey completed by NE STEM mentors after mentoring K-8 youth. B. Prompts from interview administered in person to NE STEM mentors at 12 and 24 weeks into the program. Students were not allowed to see how they had rated themselves prior. Questions 1–5 were on a scale of 1–10, 10 being the highest score. C. Prompts administered to NE STEM mentors graduating from the program (i.e., not returning the following academic year due to graduation). Questions from the end of program survey were administered to students graduating from the program immediately after their separation (±40 days)

To understand how UG mentors were evaluating their mentoring experience immediately after it had occurred, we calculated the percentage of affirmative and negative responses for the first five questions of the survey. Then, we evaluated the open-ended questions to find recurring themes and subsequently generated a rubric to further score the survey (Additional file 1: Table S1). The rubric was sent to experienced STEM faculty (external to the project) for refinement and calibrated by independently scoring surveys from UG mentors across three researchers and examined for inter-rater reliability. The rubric was then used to score the post-mentoring surveys (*n* = 64). After scoring, descriptive statistics were calculated, followed by paired, two sample *t* tests to look for significant differences in averages. Subsequently, a Pearson’s correlation analysis was used to determine what, if any, significant correlations existed.

Second, each UG mentor volunteered to be interviewed by program faculty, using a semi-structured format (Merriam 2009) after 12 and 24 weeks in the program. For the interview, students were asked to rank themselves in five categories, including organizational skills, preparedness for mentoring, STEM content knowledge, engagement skills (i.e., keeping youth engaged), and dependability, on a scale from 1 to 10 (10 being the best) (Table 3b). Students were blinded to their previous self-ranking (i.e., from 12 weeks prior). To detect changes over time, we compared 12-week ratings to 24-week ratings and determined an average for each category at each time point in order to detect any self-reported changes over time. Subsequently, we calculated the significance of these differences using Student’s *t* test.

The interview also entailed a series of open-ended questions assessing the mentor’s views of NE STEM 4U and the potential impact NE STEM 4U had on such topics as critical thinking and problem-solving skills, future teaching/mentoring, and the mentor’s likes/dislikes in the program. The specific questions asked during these interviews can be seen in Table 3b. All interviews were fully transcribed at the time they were conducted for analysis, coded, and examined for themes (Miles et al. 2014; Yin 2014).

The data associated with the end of program were collected via written exit surveys from students matriculating out of the program (*n* = 8). Data were gathered from students that were part of the program through the final semester of their senior year as well as students who decided not to participate in their final semester. Questions about career readiness and impact of NE STEM 4U on career preparation were the focus of this interview (Table 3c). We plan to conduct 5-year follow-up interviews to assess the impact of NE STEM 4U on career readiness/effectiveness. These interviews will begin in 2018.

## Results

**Fig. 3:**
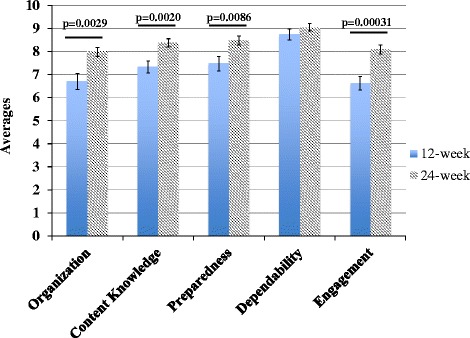
Averages of self-reported data related to organization, STEM content knowledge, preparedness to teach, dependability, and ability to engage youth from NE STEM 4U mentors. All but one of these measurements (dependability) showed significant improvement (*p* ≤ 0.05) as mentors rated themselves progressing from 12 weeks in the NE STEM 4U program to 24 weeks, *n* = 27. *Bars* represent the mean and *error bars* represent standard error. *p v*alues, using Student’s *t* test, are reported above each category that was statistically significant

In the first research question, we asked, “In what ways does an after-school outreach mentoring program for middle school students affect UG mentors in terms of personal development (development of professional skills and academic/content knowledge)?” In the post-mentoring surveys administered to all mentors at the end of the mentoring experience, a total of 94.2% of respondents indicated the experience was “beneficial to their education” (Table 4). In addition, 93.6% of mentors indicated they felt a “sense of accomplishment with helping community members” (Table 4). Mentor’s self-reported gains as a result of the NE STEM 4 U program increased over time of participation from the 12-week interview to the 24-week interview. In particular, mentors self-report of their own skills included significant gains in organization, preparedness, and engagement skills, as well as content knowledge (Fig. 3). Below are quotes from the mentors (12- and 24-week interviews) illustrating their own feelings about their personal growth:

**Table 4:** Results of post-mentoring surveys from participating undergraduate students

Prompt	Type of response	
	Affirmative	Negative
Did you find this experience to be beneficial to your education?	94.15%	5.85%^a^
Did you feel a sense of accomplishment with helping community members?	93.63%	6.37%
What would you do differently next time? (Mentors could select more than one)		
It is related to the lesson	37.63%	
It is related to classroom function	31.44%	
It is related to self-preparedness	29.38%	
It is related to the youth	17.53%	
It is related to the school	9.28%	
Other		
What did you like most about the experience? (Mentors could select more than one)		
It is related to the youth	61.88%	
It is related to the lesson	37.62%	
It is related to classroom function	15.35%	
It is related to self-preparedness	29.38%	
It is related to the school	8.42%	
Other		
What did you like least about the experience? (Mentors could select more than one)		
It is related to the youth	29.28%	
It is related to classroom function	22.65%	
It is related to the lesson	18.23%	
It is related to the school	13.26%	
Other	16.57%	

^a^Most students cited “cancellation of afterschool programming” as reasons for negative responses

One mentor said, “Definitely felt more confident (after mentoring) in STEM content as a whole”.

Another mentor indicated, regarding organizational skills, “Teaching has helped (my) organization a lot – you can’t walk into a classroom unorganized and have it go well. Teaching has helped organizational skills because others rely (on you) when committed to doing something”.

Related to core science concepts and content, mentors denoted, “(Mentoring) helped me to incorporate things that are good scientific questions.”

“(I am) very good at biology, but in other areas (TEM) lacking and so teaching has helped improve knowledge in TEM”.

Other mentors explicitly discussed how NE STEM impacted their communication skills, “Better communicator now”.

“If (I) can explain to other people, (I) can explain to patients how to use insulin effectively”.

From the interviews, 55.5% of the NE STEM 4U mentors conveyed that they would include mentoring in their careers. Additionally, 18.5% of interviewed mentors reported that their experience with NE STEM 4U had caused them to change their career trajectory to teaching science. Lastly, when UG mentors were directly asked if they intended to include teaching in their future career, 40.7% of NE STEM 4U mentors indicated that, while they did not wish to change their major to teaching, they would make a point to include teaching of some age group in their careers. Regarding changing their career to teaching, one mentor noted,

“For my career, I am now planning to include teaching at college or grad school level”.

Another mentor became aware, through NE STEM, that he/she enjoys mentoring and teaching enough to incorporate it into his/her career, “While I don’t plan to change my major to teaching, after mentoring, I do think I would like to have some aspect of teaching others in my career”.

In exit surveys, all but one (85.7%, *n* = 7 out of 8) of the student mentors matriculating out of the program stated that participating in the NE STEM 4U program directly improved their career readiness. Moreover, those mentors who were involved in curriculum planning, development, or other leadership positions highlighted the strengths they gained from serving as leaders in NE STEM 4U as well, even though they were not specifically asked a question about this aspect of the program on the exit survey. Below are a few selected quotes from the exit surveys in which mentors explicitly connect NE STEM 4U to their future, beyond their undergraduate degree:

“(NE) STEM 4U played a role in my ability to educate kids about complex material in a way that they can understand, which I think will benefit me in my future when I educate patients”.

“Well, this experience allowed (me) to make a weekly routine on the given day of teaching and I think this is a trait that is expected when one graduates, so having this extra commitment helped me gain more experience outside of taking classes”.

In the second research question, we asked, “What factors do UG mentors consider when they evaluate their experiences in an after-school mentoring program for middle school students?” Mentors voluntarily completed post-mentoring evaluations after each time they taught a lesson to middle school students. We found five common themes emerged as mentors evaluated their experience, specifically: their own content knowledge, the students’ content knowledge, reflection upon the delivery/teaching, scaffolding of the lessons for the students, and the mentor’s professional growth. Interestingly, some mentors reported that the experience helped with their own life science content knowledge, made them better teachers, or provided professional growth. However, in our analysis of these evaluations, mentors engaged in reflection of their teaching/delivery to a significantly greater extent than any of the other factors we scored (discussion of mentor content knowledge, student content knowledge, scaffolding of the lesson, or professional growth; see Table 5 for descriptive statistics). Below are a few quotes selected from the post-mentoring evaluations that relate to how preparing to teach and teaching itself were beneficial for both the mentor and mentee:

**Table 5:** Descriptive statistics for post-mentoring surveys

Scored items	Mean	Standard deviation	Minimum score possible	Maximum score possible	Minimum score achieved	Maximum score achieved
Mentor content knowledge	1.08	1.10	0	3	0	3
Student content knowledge	1.12	1.02	0	3	0	3
Reflection upon teaching/delivery	2.08	0.91	0	3	0	3
Scaffolding the lesson	1.31	0.96	0	3	0	3
Professional growth	1.25	1.37	0	3	0	3

“Today, we presented a lesson on things that I didn't know very well. In all honesty, I learned a lot of cool new information upon reading the lesson plan and preparing. It also reinforced me theory that I learn best by teaching others”.

“Some of the students seemed really indifferent to the experiment, but once I took the time to break it down and work through it with them, it went much smoother”.

“Practicing for an hour before the experiment was very helpful. We will certainly continue to do that. We felt a lot better prepared, as a team. It was very clear today that the kids learned and had a great time. They really enjoyed the lesson and were looking forward to doing it at home”.

Another mentor reflected upon how their teaching is impacting the students and that it is inspiring, “I feel like (NE) STEM is helping to inspire inquisitive young minds. I’m really hoping they pursue careers in the STEM field. Many are very smart and excited about science. I'm excited to see what the future holds for them”.

*T* test analyses (Table 6) were completed to compare the variable of reflection upon teaching/delivery to each of the other measured components (mentor content knowledge, student content knowledge, scaffolding the lessons, professional growth). Significant differences were seen between reflection upon teaching/delivery of the lesson compared to every other variable measured. These data indicate a clear impact on student self-reflection regarding their conveyance of the lesson and the level of engagement with the youth.

**Table 6:** *T* test analysis comparing reflection of lesson delivery to each measured component (mentor content knowledge, student content knowledge, scaffolding the lessons, professional growth)

Scored item 1	Scored item 2	df	*t*	p*
Reflection upon teaching/delivery	Mentor content knowledge	63	−5.657	<0.001
	Student content knowledge	63	−5.889	<0.001
	Scaffolding	63	5.671	<0.001
	Professional growth	63	3.997	<0.001

Significant differences were seen between reflection upon teaching/delivery of the lesson compared to every other variable measured

*Significant at *p* < 0.001

No other significant differences existed among the scored items on the evaluations. However, using Pearson’s correlation analysis, we observed significant correlations between how the mentors reflected upon scaffolding the lessons and three other areas: reflection upon student content knowledge, reflection upon teaching/delivery of the lesson, and reflection of their own professional growth (Table 7). No other variables showed significant correlations. These data indicated that while the mentors did reflect upon their engagement with the youth, they were unable to clearly articulate specific areas of focus of that metacognitive process.

**Table 7:** Pearson’s correlation analysis revealed significant correlations between mentors who evaluated the scaffolding of their lessons and three other areas: student content knowledge, reflection upon teaching/delivery of the lesson, and their own professional growth

Scored item	Correlation	Significance*
Student content knowledge and scaffolding lessons	0.377	0.002
Reflection upon teaching/delivery and scaffolding lessons	0.334	0.007
Professional growth and scaffolding lessons	0.279	0.026

No other variables showed significant correlation

*Significant at *p* < 0.05

When mentors were asked what they would do differently the next time they taught a lesson, three major themes emerged: increasing their self-confidence, enhancing their professional skills, and improving interactions with the primary and secondary school students. For example, regarding self-confidence, one mentor said,

“I like hearing from the after school administrators and aides that the students are learning a lot and are having a great time. The fact that the students are talking about our experiments after they leave, and are excited about them, makes me feel confident, like I am doing what I am supposed to be”.

Regarding professional development and skills for a future career, another mentor commented,

“Communication and the ability to teach are skills that can translate into a variety of fields. Honing my communication skills and figuring out how to present ideas in a way that can be understood by people of different ages and intellectual capacities will be extremely helpful in my future career”.

Regarding improving interactions with the students, mentors said,

“I think the cool lesson plans are helping to spread the excitement of STEM. Our group at ‘School X’ has gotten a lot bigger! It's really exciting to watch”.

“The kids had a lot of fun! They probably didn't even realize they were learning. Many were talking about how fun the game was as they were leaving. I'm hoping they talk to their friends about how fun STEM is and recruit more kids to join in the fun”.

It is clear that these UGs focused on how they could improve their self-confidence, enhance their professional skills, and improve their engagement and interactions with the participating youth.

## Discussion

NE STEM 4U is a pre-professional training program for undergraduates that engage socioeconomically disadvantaged youth in the community through an outreach program including STEM experiments. This study focused on the impact the UG mentors reported that NE STEM 4U had on them. While we hypothesize that the youth in the program also benefitted, this impact is beyond the scope of this study and will be presented in a subsequent, forthcoming paper. We proposed that the mentors would experience NE STEM 4U as a benefit to their education, fostering an increased sense of organization, STEM content knowledge, preparedness, dependability, and engagement. We also proposed that this program would lead to further refinement of career goals, ultimately improving their career readiness in STEM areas. Furthermore, we investigated whether participation in this program caused mentors to include teaching and mentorship in their careers.

We observed powerful affirmation from mentors that they feel this program was beneficial to their education (Table 4). Moreover, UG mentors reported improvements in personal attributes, many of which are considered important skills to make future STEM graduates employable (NACE 2014). These included engagement, dependability, organizational skills, preparedness, and STEM content knowledge (Fig. 2), with significant improvements self-reported in all areas except dependability. This may be because of all of the self-ranking categories, UGs ranked themselves, on average, the highest in terms of dependability at the early (12-week) interview, so there was not much room for increased self-rank in this area (Fig. 2).

While it may not be surprising that, in terms of evaluating their experiences immediately after mentoring, UG mentors evaluated themselves significantly more by reflecting upon their teaching/delivery of the lesson than they did in any other category (Table 6), we were intrigued by the correlations we found. UG mentors who evaluated their own scaffolding of the lessons for younger students also showed significant correlations to reflecting upon the content knowledge of students, the teaching/delivery of the lesson, and their own professional growth. It is well documented that scaffolding strategies can greatly enhance learning (Hmelo-Silver 2004; Quintana et al. 2004) for the student, so it seems consistent that mentors who reflect upon their own scaffolding of lessons would also reflect upon the younger students’ content knowledge and their own delivery of the lesson. However, the actual relationship between reflecting upon scaffolding the lesson and reflecting upon professional growth remains to be determined.

Interestingly, in their reflections, UG mentors include much information about the students, thereby implying that they really see themselves as “teachers” and the authoritative figures. Based on the reflections and interviews, this teaching/mentoring intervention through community engagement impacted many of the undergraduates’ communication skills and confidence. Grant et al. (2015) similarly found improved communication and confidence in UGs who were involved in public engagement with middle and high school students.

Peer mentoring and faculty-student mentoring have been shown previously to enhance higher-order thinking skills, build stronger relationships among undergraduates, improve career performance, and increase satisfaction in career choice (Roscoe & Chi 2007; Roscoe & Chi 2008; Malone et al. 2002). Our data lend further support and extend these results with NE STEM 4U mentors expressing the development of strong relationships across the cohort. Anecdotally, mentors cited specific benefits such as having a more veteran student available to address questions about when and how to apply for professional school or recommend the order in which they should take their biology courses.

Excitedly, the NE STEM 4U program continues to grow with an increasing number of undergraduates seeking the opportunity to participate. In the past 18 months alone, the program has grown from eight students at inception to over 60. The program grew from eight students participating year 1, to 65 year 2, to 31 this past year (this past year, many students taught more often than once weekly, thereby decreasing the number of students needed). We expect the mentor cohort to stabilize at approximately 25–35 per year.

### Limitations and areas for revised practice

In this program, first, we engaged OPS schools in socioeconomically disadvantaged areas displaying the lowest science and math scores (we did not include schools with high performance on standardized assessments). All of the youth participants are a part of a single school district, and they were assumed to have the same general background in education as their peers from a different school within the same district and geographically close by—though we understand that it is difficult to match students in terms of academic ability for intervention groups. Additionally, not all after-school time programs in the participating district run identically as there is an independent site director for each school.

Secondly, although we train all of our NE STEM mentors with the same process, we do not place limitations on the way they choose to teach. We do have overarching requirements in our program such as teach in a PBL format, complete an experiment, keep all students engaged, and do a daily assessment followed up with a long-term assessment, but we do not force all mentors to accomplish this in the same way. This can cause variations in the depth of the student participants’ comprehension of the STEM material. However, this also fosters critical thinking and encourages the independence of mentors—furthering their training in twenty-first century learning skills.

Lastly, the faculty to mentor ratio was also a challenge as we grew as a program. Specifically, this led to a high workload for the involved faculty including substantial personnel management time, on-going coordination with public school sites, management of funding, applying for additional funding, researching the effectiveness of the program, and training and certifying incoming evaluators and working with consultants for external evaluation. Providing the program during university academic year breaks also posed a challenge, leading to a revision of the program to exclude participation during winter and spring university breaks. Only seven students voluntarily participated during the summer months to prepare PBLs and materials for the following school year as well as analyze data. Therefore, as the program expands, staffing issues and staffing management will no doubt continue to be one of the greatest challenges. Retention plays a role in this challenge, too. For example, we had a mentor dropout rate of 15% with undergraduates citing lack of time for commitment to the program and the need to begin preparing for pre-professional admission exams.

## Conclusions

While more and more STEM-related departments within universities are developing community outreach programs to primary and secondary schools (James et al. 2006; Tanner et al. 2003; Williams 2002), little research has been done to investigate the impacts of mentoring on the UG mentors (Carpenter 2015). This study helps to fill that gap in the literature by providing insight into the gains the UG mentors report attaining after mentoring to middle school students. Specifically, mentors provided feedback and self-evaluation through post-mentoring reflections and interviews that revealed gains in professional training (organizational skills, communication, and preparedness), content knowledge, and engagement. Additionally, mentors reflected significantly more (*p* < 0.001) upon how they delivered the lesson than they did about their own content knowledge, the students’ content knowledge, how they scaffold the lesson, or their own professional growth in the post-mentoring reflections.

## Additional file

Additional file 1: Table S1. The following is a rubric for scoring NE STEM 4U post-mentoring surveys to gain some general insights on what mentors mention in several key areas of interest, including mentor’s content knowledge, student’s content knowledge, metacognition, scaffolding, and mentor’s experience. This rubric is intended to help quantify responses in a range represented from a score of 0, with no evidence of a particular trait to a score of 3, representing more detailed explanatory evidence. (DOC 35 kb)
