# Supplementary material for: How do undergraduate STEM mentors reflect upon their mentoring experiences in an outreach program engaging K-8 youth?
Source: Int J STEM Educ. 2017 Feb 10;4(1):3. doi: 10.1186/s40594-017-0057-4 (PMC6404414; doi:10.1186/s40594-017-0057-4)
Supplement: Supplementary file 1 — The following is a rubric for scoring NE STEM 4U post-mentoring surveys to gain some general insights on what mentors mention in several key areas of interest, including mentor’s content knowledge, student’s content knowledge, metacognition, scaffolding, and mentor’s experience. This rubric is intended to help quantify responses in a range represented from a score of 0, with no evidence of a particular trait to a score of 3, representing more detailed explanatory evidence. (DOC 35 kb) [file 40594_2017_57_MOESM1_ESM.doc]

**Supplemental Table 1**. The following is a rubric for scoring NE STEM 4U post-mentoring surveys to gain some general insights on what mentors mention in several key areas of interest, including mentors content knowledge, students content knowledge, metacognition, scaffolding, and mentor’s experience. This rubric is intended to help quantify responses in a range represented from a score of 0, with no evidence of a particular trait to a score of 3, representing more detailed explanatory evidence.

| **Criteria** | **0** | **1** | **2** | | **3** |
| --- | --- | --- | --- | --- | --- |
| Increase in Mentor’s Content Knowledge | No mention of their own content knowledge | Mentioned their own content knowledge but no increase | Mentioned an increase in their own content knowledge without going into detail | | Mentioned an increase in their own content knowledge and explained how the experience led to the positive change |
| Increase in Students’ Content Knowledge | No mention of students’ content knowledge | Mentioned students’ content knowledge but not an increase | Mentioned an increase in students’ content knowledge without going into detail | Mentioned an increase in students’ content knowledge and how the experience led to that positive change | |
| Reflection on delivery of the experience/reflection on teaching | Mentor does not mention the lesson in reflection | Mentor mentions something about the lesson | Mentions something that they thought could go better, but no mention of *how* it could go better OR something that they wanted to do to improve the lesson OR mention how he/she would change delivery of the lesson | Mentor recognizes that they could improve something about the experiment AND mention a specific example of how it could be improved | |
| Scaffolding Use | No mention of how mentor engaged with students | Mentor mentions students in the context of the lesson but gives no instructional details | Mentor discusses some instructional details of structuring the lesson for students | Mentor gives a clear and detailed description of how the lesson was structured for the students | |
| Mentor’s Professional Growth Experience in STEM | No mention of how the experience changed their professional ideas | Mention of how experience changed ideas or led to professional growth with no details mentioned | Discussed how the experience changed their ideas or led to professional growth with explanation in terms of their own self | Discussed how the experience changed ideas or led to professional growth in terms of communicating or teaching youth and/or interacting with fellow mentors (e.g. in communication skills, teamwork, prob solving) | |
